# Biodistribution of Fluorescent Albumin Nanoparticles among Organs of Laboratory Animals after Intranasal and Peroral Administration

**DOI:** 10.3390/cimb45100519

**Published:** 2023-10-11

**Authors:** Olga Morozova, Elena Isaeva, Dmitry Klinov

**Affiliations:** 1 Federal Research and Clinical Center of Physical-Chemical Medicine, Federal Medical Biological Agency, 1a Malaya Pirogovskaya Street, 119435 Moscow, Russia; 2 Ivanovsky Institute of Virology of the National Research Center of Epidemiology and Microbiology Named after N.F. Gamaleya of the Russian Ministry of Health, 16 Gamaleya Street, 123098 Moscow, Russia; 3 Department of Biological and Medical Physics, Moscow Institute of Physics and Technology, 9 Institutsky Per., 141700 Dolgoprudny, Moscow Region, Russia

**Keywords:** fluorescent BSA nanoparticles, nanoprecipitation, stability in blood sera and saliva, intranasal and peroral administration, dynamics of biodistribution in organs of mice, penetration in brain and intestine, absence of interferon gene expression induction

## Abstract

Natural, environmental and engineered nanoparticles (NP) penetrate into cells by endocytosis and induce innate immunity. The behaviour of the nanomaterials both in vitro and in vivo should be assessed. Our goal was to study protein NP stability in biological fluids and distribution in organs of animals after intranasal and oral administration. Bovine serum albumin (BSA) was labelled with the fluorescent dye RhoB and NP were fabricated by nanoprecipitation. The fluorescent protein NPwere administered intranasally and orally in laboratory-outbred mice ICR and rabbits. RhoB-BSA NP distribution in organs was detected using spectrofluorometry and fluorescent microscopy. Innate immunity was evaluated using reverse transcription with random hexanucleotide primer and subsequent real-time PCR with specific fluorescent hydrolysis probes. The labelled BSA NP were shown to remain stable in blood sera and nasopharyngeal swabs for 5 days at +37 °C. In vivo the maximal accumulation was found in the brain in 2 days posttreatment without prevalent accumulation in olfactory bulbs. For the intestine, heart and liver, the BSA NP accumulation was similar in 1 and 2 days, whereas for kidney samples even decreased after 1 day. Both intranasal and peroral administration of RhoB-BSA NP did not induce innate immunity. Thus, after intranasal or oral instillation RhoB-BSA NP were found mainly in the brain and intestine without interferon gene expression.

## 1. Introduction

Natural nanoparticles (NP) are organic or inorganic, intracellular or extracellular, with various functions. Intracellular vesicles transport both molecules, supramolecular complexes and nanoparticles from the cell surface to the interior. Over 50 proteins take part in the formation of clathrin-coated endocytic vesicles [1]. Extracellular vesicles including exosomes are released by most animal, plant and bacterial cultivable cells in vitro and some cells such as mesenchymal stem cells, B cells, endothelial, dendritic and mast cells, adipocytes, neurons, as well as all transformed tumour cells in vivo [2,3]. Lipoproteins are self-assembling structures consisting of lipids and apolipoproteins, capable of transferring lipids in both invertebrates and vertebrates. Spherical or discoidal lipoprotein NP of 7–80 nm have a core consisting of non-polar lipids, triacylglycerols and esterified cholesterol with shells from apolipoproteins, phospholipids and non-esterified cholesterol [3]. Other natural protein-based NP include lectins and ferritin. Lectins are proteins of non-immune origin able to bind specifically certain sugars on cellular membranes. Ferritin NP in bacteria, archaea and eukaryotes store iron oxides. In eukaryotes, ferritin is composed of 24 subunits with a four-helical bundle forming a hollow. Magnetotactic bacteria possess a specialized organelle—the magnetosome of 50–70 nm, consisting of lipids and containing iron-containing minerals [3].

After the first approval of NP for drug delivery in the 1990s artificial nanomaterials, spanning from inorganic and synthetic polymeric NP to protein, RNA, or lipid NP are widely used in biomedicine for prophylactic, diagnostic and therapeutic purposes, cosmetics, food processing, electronics, buildings and aeronautics. In comparison to free molecules, NP have improved bioavailability, increased intracellular delivery and important features to overcome cellular, local and systemic barriers. NP may serve as a depot for vaccine antigen storage and prolonged immune response. However, the number of NP-based drugs approved by the FDA and the European market does not correspond to the high expectations associated with nanomedicine [4,5]. The translational gap between research and clinical implementation may result from unavailable data on NP stability in living cells and organisms, distribution among organs and possible pathological sequelae. Stability, cellular uptake, accumulation in organs, biodegradation and excretion of NP in biological fluids and extracellular matrix are important for biomedical applications [6]. Despite the majority of artificial NP being biocompatible and biodegradable, some of them exhibit toxic side effects. Potential side effects and possible immunological deterioration may be caused by both NP physical sizes, shapes, and charges, as well as chemical composition and especially surface decoration [7]. Acute and chronic toxicity, biocompatibility [6,7], in vivo kinetics and the ability to escape through the reticuloendothelial system hamper the clinical implementation of NP.

Living organisms possess defense mechanisms against foreign nanomaterials including coating of NP with biocorona in biological fluids [8], cellular endocytosis with subsequent NP biodegradation in lysosomes, innate and adaptive immunity induction, reactive oxygen species (ROS) formation, neutrophil extracellular traps (NETs) and tunneling nanotubes [9,10]. Biomolecular corona on NP surfaces can be transient “soft” with high rates of both association and dissociation (in particular, a the coating consisting of albumin and fibrinogen) or “hard” with long-term stability (shells from opsonins including immunoglobulins) [8]. Surface layers consisting of biopolymers or nonionic surfactants may enhance steric hindrances and electrostatic repulsion.

Bio-reactivity, cellular uptake and distribution among organs of natural, synthetic and environmental nanomaterials including both organic and inorganic NP depend on surface proteins due to biocorona formation immediately after the addition of biorelevant media or administration into organisms. Protein conformations can be changed on the surfaces of nanostructures. Additionally, coating with foreign proteins may induce innate and adaptive immune response [9]. The surface polyethylene glycol layer does not allow intracellular delivery of nanomaterials resulting in their circulation in blood [11]. Delivery of nanomaterials to brain tumours is mediated by NP biocorona in the bloodstream; leaky blood vessels at the tumour site; enhanced penetration and retention (EPR) in transformed cells; inhibition of drug efflux in endothelial and cancer cells; and active targeting by means of specific ligands to receptors at the blood–brain barrier [12]. Thus, apolipoprotein E directs NP to the brain [13,14].

Among proteins all albumins are poorly immunogenic with induction of anti-inflammatory response in macrophages [15] and able to act as extracellular antioxidants providing competitive protection from free radicals and other harmful chemicals. Albumins provide oncotic pressure within capillaries, transport fatty acids, bilirubin, minerals and hormones, and function as anticoagulants. Many drugs and endogenous molecules are known to bind with albumins [9]. The ability of albumins of different origins to transfer drugs is used for drug delivery in nanomedicine. Albumin was the first protein approved by the Food and Drug Administration (FDA) of the USA for nanomedicines. Currently, albumin-based drugs include Abraxane (Celgene) (paclitaxel loaded albumin NP), optison (GE Healthcare, Chicago, Illinois, United States) (human serum albumin stabilized perflutren microspheres as an ultrasound contrast agent) and albumin-bound anti-cancer drugs (ABI-009 (Aadi with Celgene)—albumin-bound rapamycin and ABI-011 (NantBioScience, Los Angeles, California, United States)—albumin-bound thiocolchicine analog (IDN 5405)) [9].

Biodistribution and biodegradation of foreign NP depend on the protection systems of organisms including both innate and subsequent adaptive immune response. Inflammation is a key reaction following exposure to any foreign solid material, including NP. Induction of pro-inflammatory cytokines was shown for several nanomaterials in vitro and some cytokines can bind to nanomaterials [9,16].

Oxidative stress is responsible for NP-induced injury [17]. The kidney is susceptible to reactive oxygen species (ROS)-induced injury [17].

Non-invasive painless delivery of nanomedicines across the biological barriers of the mucosal surfaces may induce innate and adaptive systemic and mucosal immunity to prevent respiratory and intestinal infections. At present oral administration is considered preferable in comparison with other routes of NP administration including intravenous and intranasal.

Our research was aimed at the protein NP stability and distribution in organs of mice and rabbits after non-invasive intranasal and peroral administrations.

## 2. Materials and Methods

### 2.1. Fluorescent Protein NP Fabrication

Fluorescent rhodamine dye RhoB was used to label the protein—the bovine serum albumin (BSA) in order to construct fluorescent protein NP. The rhodamine dyes are more stable in cellular compartments [9] compared to fluorescein derivatives and cyanine dyes. Fluorescence emission with a maximum at 580 nm at an excitation wavelength of 550 nm is typical for the fluorescent rhodamine dye RhoB and far from the autofluorescence of phenyl alanine (λ max = 282 nm), tyrosine (λ max = 303 nm) and tryptophane (λ max = 348 nm) amino acid residues of proteins from living cells. Therefore, the fluorescence emission spectra of the rhodamine dye RhoB and cellular proteins do not overlap.

BSA (Sigma-Aldrich, St. Louis, MO, USA) was labeled with the rhodamine dye RhoB (Sigma-Aldrich, USA) in 0.1 M Na_2_CO_3_ pH 9.3 overnight and purified by Sephadex G25 gel chromatography with mild centrifugation at 700× *g* for 3 min at room temperature. The RhoB/BSA molar ratio did not exceed 5 and permitted to retain conformational stability of the fluorescent protein [18].

The fluorescent protein NP were constructed without cross-linking using nanoprecipitation of the pre-labeled protein solution in fluoroalcohol as previously described [18]. In brief, 10 mg/mL RhoB-BSA was dissolved in 1,1,1,3,3,3-Hexafluoro-propan-2-ol (HFIP) (Sigma-Aldrich (USA)). Then, the fluorescent protein solution in HFIP was added dropwise to 40% ethanol in water (1/10 part of the total volume resulting in the final protein concentration of 2 mg/mL). Permanent vigorous stirring was necessary to maintain the stability of the three-phase system including two solvents (water and HFIP) and one anti-solvent (ethanol). The mixture was immediately placed at 58.2 °C (HFIP boiling point). To accelerate evaporation of both alcohols the nanoprecipitation was performed under pressure less than 25 mBar. Alcohol evaporation time depended on both the pressure and volume of the mixture. Then, water-insoluble particles were pelleted at 15,000 g and washed with deionized water 3 times to remove the residual protein molecules. An alternate way of NP purification was gel chromatography using Sephadex G200 with subsequent centrifugation at 700× *g*. Microparticles were removed by additional differential centrifugation at 700–1000× *g*, leaving the NP in the supernatant.

Concentrations of BSA and RhoB were measured using the spectrophotometer NanoDrop 2000c UV-Vis (Thermo Scientific, Waltham, MA, USA) and calculated on the base of the molar extinction coefficient ε = 43,824 M^−1^ cm^−1^ (at λ = 280 nm) for BSA and ε = 75,976 M^−1^ cm^−1^ (at λ = 560 nm) for RhoB.

RhoB-BSA NP hydrodynamic radii were measured by dynamic light scattering (DLS) using NANO-flex 180° (Microtrac, York, PA, USA).

### 2.2. Animals

Mice ICR (30 males, 10–12 g) and outbred rabbits (5 males) were obtained from the animal nursery “Andreevka”, Moscow region, Russia. Light ether anaesthesia was used for the experiments with the laboratory animals. Ethical approval from the Ethics Committee of the National Research Center of Epidemiology and Microbiology of N.F. Gamaleya of the Russian Ministry of Health, Moscow, Russia was taken before starting the research (Approval Code # 18 2022-02-21).

### 2.3. Intranasal and Peroral Instillation of the Fluorescent Protein NP in Mice

RhoB-BSA NPwere administered intranasally to 10 mice and 2 rabbits, and perorally to other groups consisting of 10 mice and 2 rabbits. Two control groups from 10 mice or 1 rabbit received normal PBS buffer. Laboratory animals under light ether anaesthesia for a few minutes in a horizontal position with their heads slightly below their bodies were administered the fluorescent protein NP. For intranasal instillation 50 μL RhoB-BSA NP in deionized water with a final protein concentration of 10 mg/mL per each mouse and 200 μL per rabbit were added dropwise via nose using standard laboratory micropipette with aerosol tips. For peroral procedures, 100 μL RhoB-BSA NP in deionized water per mouse and 500 μL per rabbit were added dropwise via mouth using a standard laboratory micropipette with aerosol tips. The experimental animals should be kept in a horizontal position for several minutes after intranasal and oral NP administration.

### 2.4. Spectrofluorometry

Fluorescence emission with a maximum at 580 nm at an excitation wavelength of 550 nm was daily measured for 10% homogenates of organs of mice in PBS after intranasal and peroral administration in dynamics using spectrofluorometer “Fluoromax+” (Horiba Scientific, Kyoto, Japan). Statistical calculations of average values and standard deviations of the RhoB fluorescence emission measured in relative fluorescent units (rfu) were performed using Microsoft Excel 2016 software.

### 2.5. Fluorescent Microscopy

Histological sections and prints of the mouse and rabbit organs in 2 days after intranasal and peroral administration of RhoB-BSA NP on glass slides were fixed with cold 4% formaldehyde solution in PBS. After washing with cold PBS 3 times the cell DNA was strained with the fluorescent intercalating dye Hoechst 33342 (Abcam, Waltham, MA, USA).

The fluorescent analysis was performed using the microscope Nikon Eclipse Ti (Nikon, Minato City, Tokyo, Japan) with a set of filters providing excitation/emission for RhoB (528–553 nm/590–650 nm) and for Hoechst 33342 (340–380 nm/435–485 nm).

### 2.6. Cytokine Gene Expression

RNA of mouse interferons (IFN)α, β, γ, λ, interleukins (IL)4, IL10, IL12 and IL17 were measured using reverse transcription with random hexanucleotide primer and subsequent real-time PCR (RT^2^-PCR) with cytokine-specific fluorescent hydrolysis probes as previously described [9].

## 3. Results

### 3.1. Stability of RhoB-BSA NP in Biological Fluids

**Figure 1:**
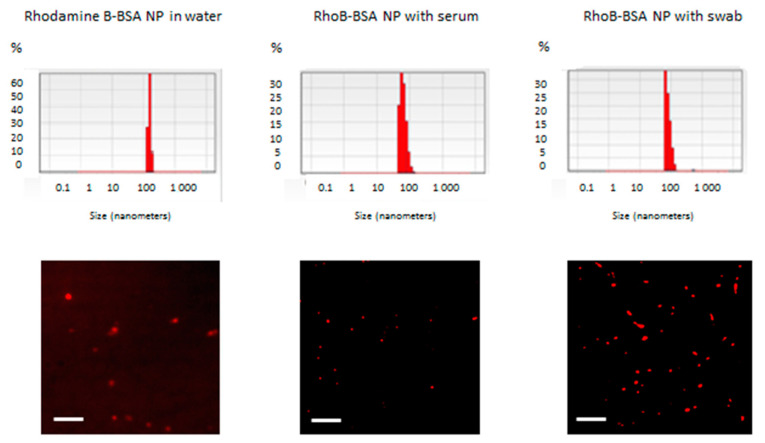
Stability of RhoB-BSA NP after incubation in the presence of human blood serum or nasopharyngeal swab at +37 °C for 5 days. Upper row shows the dynamic light scattering (DLS) data and permits to estimate mean hydrodynamic radii of the RhoB-BSA NP. Axis X stands for sizes (nm), axis Y—% of RhoB-BSA NP of corresponding sizes. Lower row corresponds to the fluorescent microscopy images. Scale bar is 2 μm in each image.

The fluorescent protein were constructed by nanoprecipitation of RhoB-labelled BSA as earlier described [18] and analyzed using dynamic light scattering and fluorescent microscopy (Figure 1).

Hydrodynamic radii of the RhoB-BSA NP in deionied water immediately after the nanoprecipitation (Figure 1, left panel) and after incubation with adult human blood serum and nasopharyngeal swab during 5 days were similar (nearly 100 nm) (Figure 1). The fluorescent microscopic images (Figure 1, low row) confirmed the stability of the RhoB-BSA NP in the presence of biological fluids in vitro.

RhoB-BSA NP remained stable during storage in water for several years [9,18] and after incubation with blood sera and saliva for several days at +37 °C (Figure 1). Fetal calf serum without a complement system and adult human blood sera resulted in similar stability estimations. Moreover, the presence of nasopharyngeal swabs also did not change BSA-NP size ranges (Figure 1).

### 3.2. Distribution of RhoB-BSA NP among Mouse Organs after Intranasal and Oral Administration

**Figure 2:**
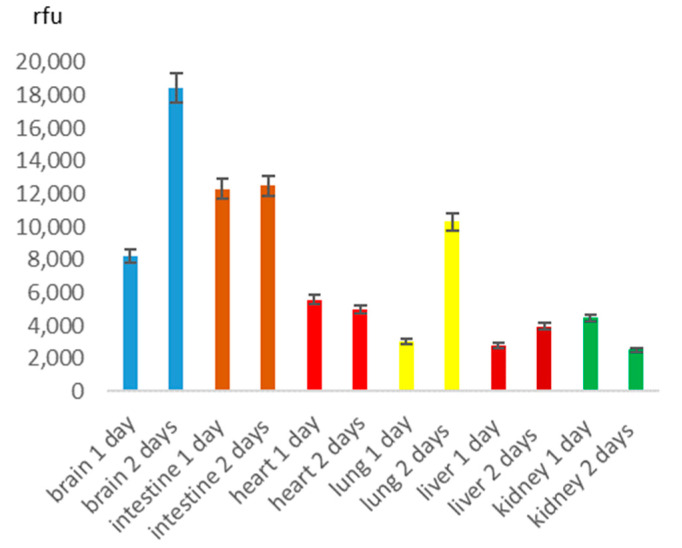
Dynamics of RhoB-BSA NP accumulation in different organs of ICR mice after intranasal administration. Axis Y corresponds to the relative fluorescent units (rfu).

Dynamics of BSA NP distribution in the various mouse organs on the base of spectrofluorometry measurements (an excitation wavelength 550 nm, an emission at 580 nm) were not similar for different organs of mice after mucosal instillation of the RhoB-BSA NP (Figure 2). The maximal values for brain samples after both intranasal and peroral administration of the protein were in 2 days posttreatment as well as for mouse lung samples (Figure 2) and cell lines [9]. For the intestine, heart and liver, the BSA NP accumulation was approximately similar in 1 and 2 days after administration whereas for kidney samples the fluorescence emission of the RhoB-BSA NP even decreased in 1 day posttreatment (Figure 2). Evident variations between intranasal and peroral administration ways as well as among animals in each group were observed.

**Figure 3:**
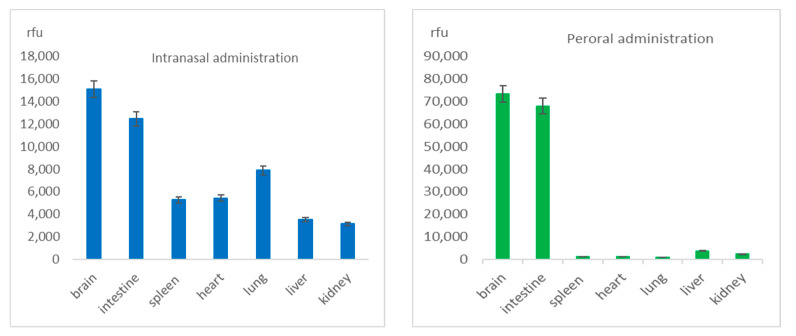
Distribution of RhoB-BSA NP among organs of laboratory mice ICR in 2 days after intranasal and peroral administration on the base of spectrofluorometry measurements of 10% homogenates of individual mouse organs and subsequent calculations of average values. Axis Y corresponds to the relative fluorescent units (rfu).

**Figure 4:**
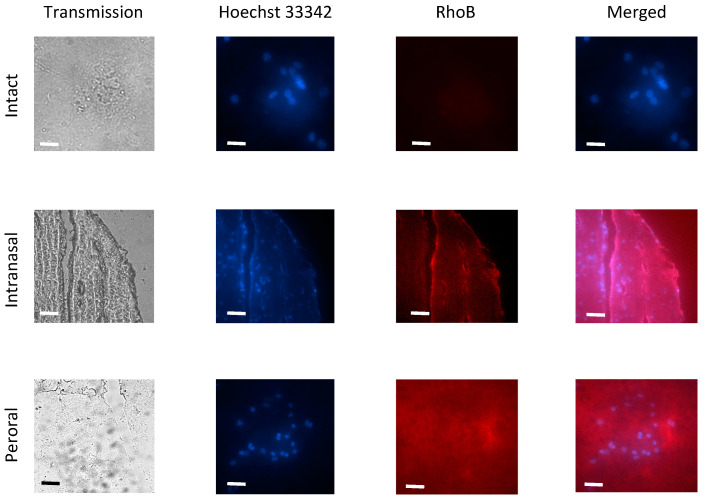
Penetration of RhoB-BSA NP in mouse brain in 2 days after intranasal and peroral administration. Scale bar is 20 μm in each image.

Biodistribution of the RhoB-BSA NP among organs of the laboratory mice was similar in 2 days after both intranasal and peroral administration (Figure 3 and Figure 4).

After both ways of the mucosal instillation, the RhoB-BSA NP could reach the brain through axonal transport along the olfactory nerve as shown by fluorescent microscopy (Figure 4) which confirmed our data of spectrofluorometry (Figure 2 and Figure 3). The fluorescent protein NP could be found both within brain cells and in the extracellular matrix (Figure 4).

**Figure 5:**
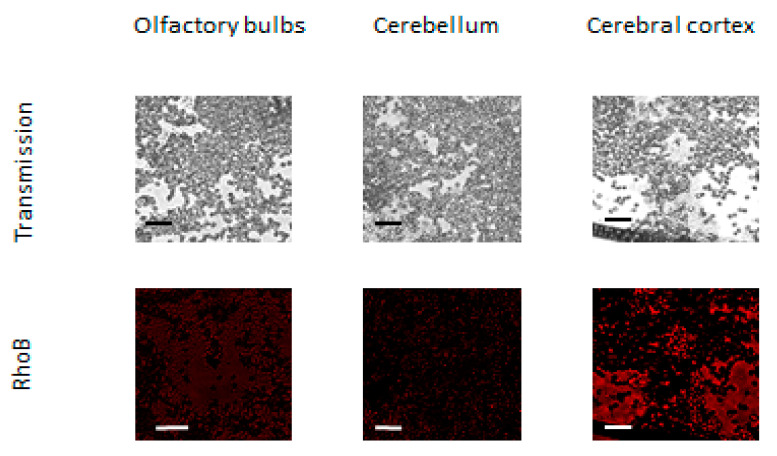
Rho B-BSA NP distribution in different parts of rabbit brain in 2 days after intranasal administration. Scale bar is 20 μm in each image.

Fluorescent microscopy of the RhoB-BSA NP distribution in different parts of the rabbit brain did not reveal their selective accumulation only in the olfactory bulbs where BBB is absent (Figure 5). Fluorescent emission was also registered in the brain hemispheres and cerebellum (Figure 5).

### 3.3. Cytokine Gene Expression Analysis after Intranasal and Oral Administrations of RhoB-BSA NP

Endocytosis-mediated cellular uptake of RhoB-BSA NP lacking any known immunostimulatory component causes their proteolytic degradation in lysosomes with BSA peptides presentation and possible induction of innate immunity. Cytokine gene expression was estimated in 1 day after RhoB-BSA NP intranasal and peroral administration by means of RT^2^-PCR. RNA of IFN of I, II and III types, as well as interleukins IL12 and 17, were found neither in the mouse blood leukocytes nor in sera. Only IL4 and IL10 RNA were detected in one of 10 mice for each.

## 4. Discussion

BSA, a globular non-glycosylated protein with a molecular weight close to 66,430 Da, was used for NP fabrication due to its high solubility in water and fluoroalcohols as well as stability in a wide range of pH (4–9) and temperatures (up to 60 °C for 10 hours).

To monitor inorganic nanomaterials and especially metallic NP a number of methods have been developed and widely used whereas artificial protein NP remain undetectable within living cells and cannot be isolated because of ubiquitous natural solid NP and vesicles. Therefore, fluorescent labelling was necessary to reveal the biodistribution of BSA NP among organs of laboratory animals by using quantitative spectrofluorometry and fluorescent microscopy. The rhodamine dyes are stable in a wide range of pH in the presence of high concentrations of proteins. RhoB fluorescence emission maximum at 580 nm is far from the autofluorescence of aromatic amino acid residues of proteins from living cells. Consequently, the fluorescence emission spectra of the rhodamine dye RhoB and cellular proteins do not overlap. To reduce the risk of conformational changes of BSA, the molar ratio RhoB/BSA did not exceed 5. BSA conformational and antigenic stability was confirmed by ELISA with polyclonal antibodies against BSA as earlier described [18].

Validation of the spectrofluorometric and microscopic methods with necessary controls including the fluorescent dye RhoB solution with known concentrations and molar extinction coefficient, the fluorescent protein RhoB-BSA and the corresponding nanoparticles were previously described in detailsed [9,18]. Despite covalent binding between BSA and RhoB during fluorescent labelling and subsequent gel chromatography purification, the free dye molecules cannot be completely excluded from consideration because of possible degradation of the RhoB-BSA inside cells [9]. Autofluorescence of organs of intact mice and rabbits in the range near 580 nm was minimal as shown by means of fluorescent microscopy (Figure 4).

RhoB-BSA were stable in water at +4 °C during the whole period of observation for 3 years [9,18], whereas in the presence of human or fetal bovine blood sera and nasopharyngeal swabs with saliva for 5 days at +37 °C (Figure 1); however, they were hydrolyzed with trypsin [18]. In addition to high total protein concentrations in blood and possible competitive inhibition between blood proteins and the fluorescent protein NP, protease inhibitors encompassing nearly 10% of all blood proteins may be responsible for the NP stability [19]. Most inhibitors are highly specific for single or several related proteinases whereas the α-2-macroglobulin and the alpha 1-proteinase (α-1-antitrypsin) have a broader specificity [19]. Mucosal swabs and saliva also contain a number of proteins, including mucin, histatin, cystatin, statherin, amylase, lingual lipase, secretory immunoglobulins and proline-rich protein [20]. These proteins maintain tooth and mucosal integrity, mucosal immunity and are involved in digestion, lubrication, buffering and antibacterial activity [20]. Complex regulation of proteins with enzymatic, immunomodulation and antimicrobial properties in saliva is mediated by a balance between proteases mostly derived from white blood cells and bacteria in the oral cavity and their inhibitors. Protease inhibitors of saliva include Kunitz inhibitors, serpins and cystatins. Salivary protease inhibitory proteins include antileukoproteinase and cysteine protease inhibitors, cystatin-B, -C, -D, -S, -SA and –SN [20]. All the secreted protease inhibitors may determine protein NP stability in the presence of mucosal swabs (Figure 1). Limited stability of BSA NP during incubation in blood sera and with swabs and saliva for several days at +37 °C (Figure 1) can be caused by from high total protein concentrations in NP in comparison with available proteases in the biological fluids, competitive inhibition of protease activity with other proteins derived from the biological samples, protease inhibitors in blood and saliva [19,20] and slow layer-by-layer degradation of surface proteins in NP with possible induction of innate immunity [9,10]. Available data on the stability of protein NP in vitro [9,18] (Figure 1) permit to suggest their implementation as diagnostic tools and potential drug delivery vehicles.

RhoB-BSA NP detection, quantitation, biodistribution and degradation in living organisms in vivo and in eukaryotic cells in vitro [9] were based on quantitative spectrofluorometry and fluorescent microscopy since direct visualization of unlabeled protein NP or their isolation from cells are hardly possible due to ubiquitous natural solid NP and vesicles. However, the stability of the fluorescent rhodamine dye RhoB in lysosomes in an acidic environment and oxidation in the presence of ROS cannot be excluded from consideration. Similar gradual uptake of the RhoB-labelled BSA NP in human immortal tissue cultures and primary blood leukocytes for 2 days and following decline up to 5 days allowed us to suggest that intracellular delivery and biodegradation of the protein NP are based on common mechanisms [9]. The maximal levels of the RhoB-BSA NP were detected in 1–2 days after both intranasal or oral instillation (Figure 2) and coincided with the dynamics of their cellular uptake in vitro [9]. However, after a single intravenous injection via the mouse tail vein, two different protein-based NP rapidly transferred within 15 min in lung-associated lymph nodes [21]. More than half (57–73%) of these protein NP excreted in 24 h after intravenous administration [21]. Evidently, noninvasive mucosal instillations showed slower dynamics of the protein NP biodistribution (Figure 2 and Figure 3) compared to intravenous injections [21] due to the required additional stage of NP penetration into blood or lymph.

Protein-based NP extensively translocated throughout different mouse organs and tissues after mucosal instillations via nose or mouth (Figure 2 and Figure 3) and after intravenous injection [21]. The protein-based were found in the circulatory and lymphatic endothelial cells, pulmonary epithelial cells, the interstitium of the lung, the outer capsule and perilymphoid zones of the spleen, the sinusoids of the liver, the convoluted tubules of the kidney, and draining lymph nodes [21]. The broad distribution of protein NP among different mouse organs and tissues suggested their translocation from the vascular system to interstitial areas, in the lymphatics, and interstitial areas of organs [21]. Similarly, RhoB-BSA NP after intranasal administration rapidly traversed the airway epithelium and entered in endothelial cells of blood vessels by means of transcytosis via cells or through intercellular tight junctions. Surprisingly, after intravenous administration, the protein-based were found in all organs, except the brain [21], whereas after intranasal and oral administration the RhoB-BSA NP accumulation significantly differed for different organs with prevalent accumulation in the brain (Figure 2 and Figure 3). The gelatin NP appeared to accumulate in macrophages, thus reaching macrophage-rich organs and crossing the blood–brain barrier (BBB) [22].

Whereas microparticles >2000 nm accumulate within the spleen, liver, and lung capillaries, NP of 100–200 nm could escape filtration by the liver and spleen. The broad distribution of the fluorescent protein NP in mouse organs (Figure 2 and Figure 3) [21,22], relative stability for a few days (Figure 2), penetration into the circulatory system after mucosal administration and absence of accumulation in liver and spleen (Figure 2 and Figure 3) may serve as evidence of the NP escape from reticuloendothelial system in spite of mucosa-associated lymphoid tissue (MALT) is known as an important part of the system.

Prevalent accumulation of BSA NP in the brain could be caused by penetration through olfactory bulbs where BBB is absent. BBB has the least permeable capillaries due to intercellular tight junctions. Therefore, the BBB is the obstacle or a rate-limiting factor for the intracerebral delivery of drugs [23]. Currently, most drugs, including recombinant proteins, antibodies or other biopolymers, are not capable of crossing the BBB. Small molecules of molecular weight < 600 Da including peptides with less than 6 amino acid residues and hydrophobic molecules soluble in lipids can penetrate the brain. The BBB also restricts more than 98% of small drugs. Nasal or transcranial administration, infused hypertonic agents, and lipidation of small-molecule drugs were used to transfer medicines through the BBB [23]. Free diffusion across the BBB is known for several liposoluble small molecules (MW < 400 Da). The chemical methods to overcome BBB include: (1) chemical modification of the drug to form a more lipophilic prodrug; (2) coupling the drugs with mannitol or aromatic substances (such as borneol and musk), because mannitol could induce a high osmotic pressure to opening the BBB temporarily and aromatic substances could cross the BBB as the resuscitation medicine; (3) using appropriate chemical drug delivery system or drug carrier with the ability to cross BBB. Protein NP were suggested to overcome the limitations of free low-molecular-weight drugs and to cross systemic, microenvironmental and cellular barriers.

Although the majority of large blood molecules were prevented from penetration into the brain by the tight junctions in the BBB, the specific and, nonspecific transcytotic mechanisms exist to transport large molecules and complexes across the BBB. The passive penetrations in brains inside leukocytes and the transcellular lipophilic pathway common for lipid agents could be excluded from mechanisms of RhoB-BSA NP localization in the brain. Mechanisms of active NP uptake such as tight junction modifications and modulation, carrier-mediated transcytosis and receptor-mediated transcytosis seem unlikely. Consequently, the protein NP adsorption and unspecific endocytosis with subsequent transcytosis may be responsible for the RhoB-BSA NP distribution in different parts of mouse and rabbit brains (Figure 4 and Figure 5).

Nanocarriers can favour the delivery of chemotherapeutics to brain tumours owing to different strategies, including chemical stabilization of the drug in the bloodstream; passive targeting (because of the leaky vascularization at the tumour site); inhibition of drug efflux mechanisms in endothelial and cancer cells; and active targeting by exploiting carriers and receptors overexpressed at the blood–brain tumour barrier. Within this concern, a suitable nanomedicine-based therapy for gliomas should not be limited to cytotoxic agents, but also target the most important pathogenetic mechanisms, including cell differentiation pathways and angiogenesis. Moreover, the combinatorial approach of cell therapy plus nanomedicine strategies can open new therapeutical opportunities. The major part of attempted preclinical approaches on animal models involves active targeting with protein ligands, but, despite encouraging results, a small number of nanomedicines reached clinical trials, and most of them include drug-loaded nanocarriers free of targeting ligands, also because of safety and scalability concerns.

BBB breakdown in vivo by altering the permeability of both cell membranes and intercellular tight junctions for foreign NP could induce brain edema formation [24]. oteworthy that the leakage of Evans blue dye from NP was observed largely in the ventral surface of the brain and the proximal frontal cortex but the dorsal surfaces of the cerebellum showed mild to moderate staining [24].

Detection of the fluorescent protein NP in the intestine was not self-evident due to mucosal barriers after oral and nasal instillations, a large pH gradient ranging from pH 1–2.5 in the stomach to pH 7–8 in the colon, proteolytic enzymes (pepsin in the stomach as well as trypsin and chymotrypsin in the duodenum) [25], tight junctions between neighboring epithelial cells, the enterocytes of the intestinal epithelium and the subepithelial tissue [26]. The fluorescent rhodamine dye RhoB appeared to remain stable in the intestine (Figure 2 and Figure 3) despite the aggressive conditions of the gastrointestinal tract. The weak innate immunity induction after intranasal and peroral RhoB-BSA NP administration did not correspond to Th1 polarized innate immunity in human cell lines and blood mononuclear cells [9] probably due to minimal amounts of NP in blood and prevalent accumulation in the brain and intestine with isolated immunity systems of the central nervous system and mucosa. Taken together, intranasal and peroral administration of RhoB-BSA NP did not induce systemic innate immunity that suggested negligible specific cellular and humoral response. Therefore, the fluorescent rhodamine RhoB-BSA NP elimination from living organisms could not be mediated by the immune system.

## 5. Conclusions

Protein NP fabricated by nanoprecipitation without a cross-linking were stable during a few years in water in a refrigerator and for several days in biological fluids in physiological conditions. After intranasal or oral administration they accumulated mainly in the brain and intestine with peak values in 1–2 days. Innate immune response in mice was weak (if any). Transfer of protein NP through BBB may be used for targeted drug delivery in the central nervous system.

## Data Availability

Data supporting reported results are available on request.

## References

[1] Kaksonen M., Roux A. (2018). Mechanisms of clathrin-mediated endocytosis. Nat. Rev. Mol. Cell Biol..

[2] Tian J., Casella G., Zhang Y., Rostami A., Li X. (2020). Potential roles of extracellular vesicles in the pathophysiology, diagnosis, and treatment of autoimmune diseases. Int. J. Biol. Sci..

[3] Stanley S. (2014). Biological nanoparticles and their influence on organisms. Curr. Opin. Biotechnol..

[4] Bobo D., Robinson K.J., Islam J., Thurecht K.J., Corrie S.R. (2016). Nanoparticle-based medicines: A review of FDA-approved materials and clinical trials to date. Pharm. Res..

[5] Anselmo A.C., Mitragotri S. (2019). Nanoparticles in the clinic: An update. Bioeng. Transl. Med..

[6] Schubert J., Chanana M. (2018). Coating Matters: Review on Colloidal Stability of Nanoparticles with Biocompatible Coatings in Biological Media, Living Cells and Organisms. Curr. Med. Chem..

[7] Duan J., Liang S., Feng L., Yu Y., Sun Z. (2018). Silica nanoparticles trigger hepatic lipid-metabolism disorder in vivo and in vitro. Int. J. Nanomed..

[8] Cedervall T., Lynch I., Lindman S., Berggård T., Thulin E., Nilsson H., Dawson K.A., Linse S. (2007). Understanding the nanoparticle-protein corona using methods to quantify exchange rates and affinities of proteins for nanoparticles. Proc. Natl. Acad. Sci. USA.

[9] Morozova O.V., Sokolova A.I., Pavlova E.R., Isaeva E.I., Obraztsova E.A., Ivleva E.A., Klinov D.V. (2020). Protein nanoparticles: Cellular uptake, intracellular distribution, biodegradation and induction of cytokine gene expression. Nanomedicine.

[10] Morozova O.V. (2023). Mechanisms of cellular protection against nanomaterials. Mol. Med..

[11] Niidome T., Yamagata M., Okamoto Y., Akiyama Y., Takahashi H., Kawano T., Katayama Y., Niidome Y. (2006). PEG-modified gold nanorods with a stealth character for in vivo applications. J. Control. Release.

[12] Ferraris C., Cavalli R., Panciani P.P., Battaglia L. (2020). Overcoming the blood-brain barrier: Successes and challenges in developing nanoparticle-mediated drug delivery systems for the treatment of brain tumours. Int. J. Nanomed..

[13] Kreuter J., Shamenkov D., Petrov V., Ramge P., Cychutek K., Koch-Brandt C., Alyautdin R. (2002). Apolipoprotein-mediated transport of nanoparticle-bound drugs across the blood-brain barrier. J. Drug Target..

[14] Michaelis K., Hoffmann M., Dreis S., Herbert E., Alyautdin R., Michaelis M., Kreuter J., Langer K. (2006). Covalent linkage of apolipoprotein E to albumin nanoparticles strongly enhances drug transport into the brain. J. Pharmacol. Exp. Ther..

[15] Dutta D., Sundaram S.K., Teeguarden J.G., Riley B.J., Fifield L.S., Jacobs J.M., Addleman S.R., Kaysen G.A., Moudgil B.M., Weber T.J. (2007). Adsorbed proteins influence the biological activity and molecular targeting of nanomaterials. Toxicol. Sci..

[16] Aljabali A.A., Obeid M.A., Bashatwah R.M., Serrano-Aroca Á., Mishra V., Mishra Y., El-Tanani M., Hromić-Jahjefendić A., Kapoor D.N., Goyal R. (2023). Nanomaterials and Their Impact on the Immune System. Int. J. Mol. Sci..

[17] Makhdoumi P., Karimi H., Khazaei M. (2020). Review on Metal-Based Nanoparticles: Role of Reactive Oxygen Species in Renal Toxicity. Chem. Res. Toxicol..

[18] Morozova O.V., Pavlova E.R., Bagrov D.V., Barinov N.A., Prusakov K.A., Isaeva E.I., Podgorsky V.V., Basmanov D.V., Klinov D.V. (2018). Protein nanoparticles with ligand-binding and enzymatic activities. Int. J. Nanomed..

[19] Bodmer J.L., Schnebli H.P. (1984). Plasma proteinase inhibitors. Schweiz. Med. Wochenschr..

[20] Yamamoto K., Hiraishi M., Haneoka M., Fujinaka H., Yano Y. (2021). Protease inhibitor concentrations in the saliva of individuals experiencing oral dryness. BMC Oral Health.

[21] Kaiser C.R., Flenniken M.L., Gillitzer E., Harmsen A.L., Harmsen A.G., Jutila M.A., Douglas T., Young M.J. (2007). Biodistribution studies of protein cage nanoparticles demonstrate broad tissue distribution and rapid clearance in vivo. Int. J. Nanomed..

[22] Hong S., Choi D.W., Kim H.N., Park C.G., Lee W., Park H.H. (2020). Protein-Based Nanoparticles as Drug Delivery Systems. Pharmaceutics.

[23] He Q., Liu J., Liang J., Liu X., Li W., Liu Z., Ding Z., Tuo D. (2018). Towards improvements for penetrating the blood-brain barrier-recent progress from a material and pharmaceutical perspective. Cells.

[24] Sharma H.S., Hussain S., Schlager J., Ali S.F., Sharma A. (2010). Influence of nanoparticles on blood-brain barrier permeability and brain edema formation in rats. Acta Neurochir. Suppl..

[25] Vitulo M., Gnodi E., Meneveri R., Barisani D. (2022). Interactions between Nanoparticles and Intestine. Int. J. Mol. Sci..

[26] Lundquist P., Artursson P. (2016). Oral absorption of peptides and nanoparticles across the human intestine: Opportunities, limitations and studies in human tissues. Adv. Drug Deliv. Rev..

